# A novel and practical asymmetric synthesis of dapoxetine hydrochloride

**DOI:** 10.3762/bjoc.11.283

**Published:** 2015-12-17

**Authors:** Yijun Zhu, Zhenren Liu, Hongyan Li, Deyong Ye, Weicheng Zhou

**Affiliations:** 1 State Key Lab of New Drug & Pharmaceutical Process, Shanghai Key Lab of Anti-Infectives, State Institute of Pharmaceutical Industry, No. 285, Gebaini Rd., Shanghai 201203, China; 2 School of Pharmacy, Fudan University, No. 826, Zhangheng Rd., Shanghai 201203, China

**Keywords:** asymmetric synthesis, dapoxetine hydrochloride, stereoselectivity, (S)-*tert*-butanesulfinamide

## Abstract

A novel and practical asymmetric synthesis of dapoxetine hydrochloride by using the chiral auxiliary (*S*)-*tert*-butanesulfinamide was explored. The synthesis was concise, mild, and easy to perform. The overall yield and stereoselectivity were excellent.

## Introduction

**Figure 1:**
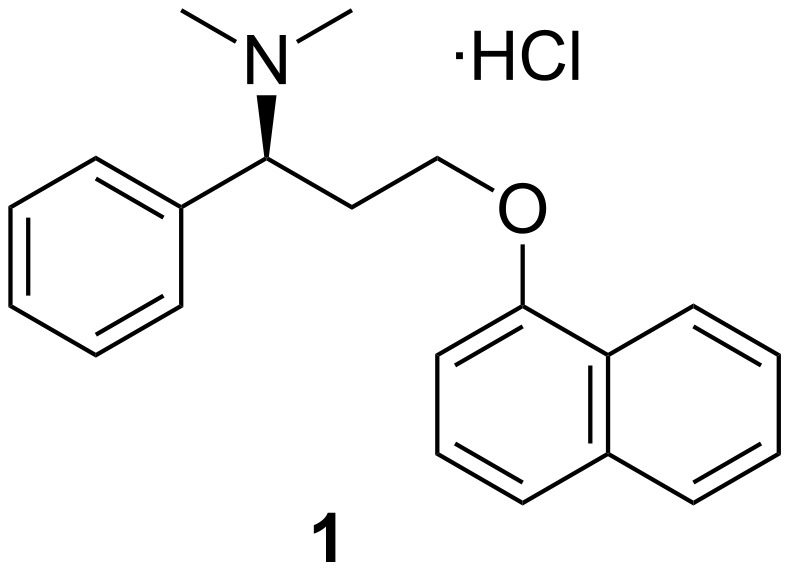
Dapoxetine hydrochloride (**1**).

Premature ejaculation (PE) is the most frequent form of ejaculatory dysfunction with a distribution of 39% of the general male population [1–2]. Dapoxetine hydrochloride (**1**, (*S*)-(+)-*N,N*-dimethyl-[3-(naphthalen-1-yloxy)-1-phenylpropyl]amine hydrochloride, Figure 1) was approved by EMA in 2009 for the special treatment of PE [3–4]. By virtue of its fast acting property and rapid elimination from the body, it is one of the more effective and safe drugs for treating PE.

For this reason, the synthesis of this interesting drug has attracted great attention, especially asymmetric synthesis approaches. However, only a few methods have been reported for the synthesis of enantiopure dapoxetine hydrochloride. The earlier methods included chiral/enzymatic resolution [5], whereas the newer approaches encompass asymmetric dihydroxylation of *trans*-methyl cinnamate or cinnamyl alcohol [6], chiral azetidin-2,3-dione [7], asymmetric C–H amination reactions of a prochiral sulfamate [8], oxazaborolidine reduction of 3-chloropropiophenone or ketone [9], and an imidazolidin-2-one chiral auxiliary mediated acetate aldol reaction [10]. However, these methods are undermined by poor yield, low enantioselectivity, and complex synthetic procedure.

Chiral *tert*-butanesulfinamide, developed by García Ruano and Ellman, has been proven to be a broadly useful reagent for the preparation of chiral amines via the chiral *N*-*tert*-butanesulfinylimine intermediates [11–12]. Due to its high diastereoselectivity and convenient cleavage of the *N*-*tert*-butanesulfinyl group, it has become an excellent chiral auxiliary in the synthesis of chiral amine compounds [13]. This work was devoted to develop an efficient synthetic route for the synthesis of (*S*)-dapoxetine (**1**) through this chiral auxiliary.

## Results and Discussion

**Scheme 1:**
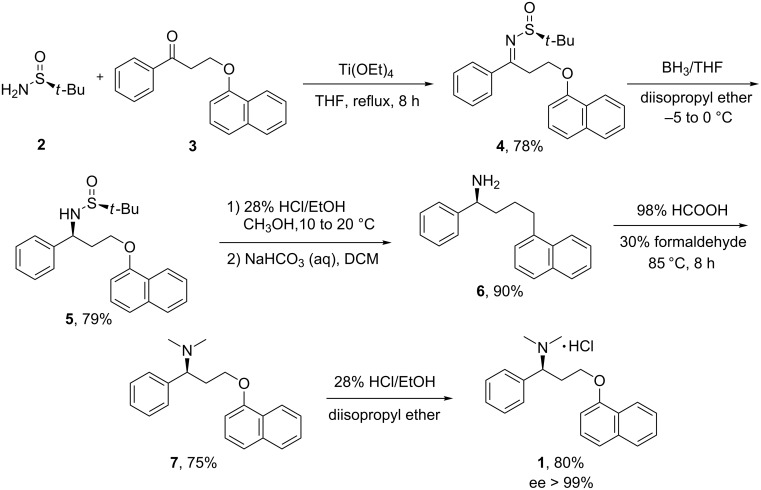
Asymmetric synthesis of **1**.

Herein, a novel and practical synthesis of **1** (Scheme 1) based on (*S*)-*tert*-butanesulfinamide (**2**) was developed.

3-(Naphthalen-1-yloxy)-1-phenylpropan-1-one (**3**), which was commercially available from J&K Chemical Ltd., was chosen as a key building block to be condensed with **2** to form the imine. The reaction in the presence of Ti(OEt)_4_ gave compound **4** in 78% yield [14] (Scheme 1).

**Scheme 2:**
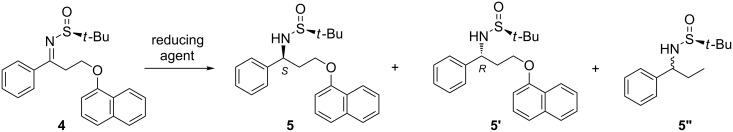
Reduction of sulfinylimine **4**.

The diastereoselective reduction of imine **4** (Scheme 2) was the key step in this route. Accordingly, various conditions were screened and the results are presented in Table 1.

**Table 1:** Conditions for the reduction of sulfinylimine **4**.

entry	reductant	solvent	*T* (°C)	time (h)	crude product (**5:5’:5”**)	de^a^ (%)

1	NaBH_4_ (0.8 equiv)	THF	25	1	28%:9%:62%	51
2	NaBH_4_ (0.8 equiv)	THF	−30	3	56%:11%:33%	67
3	NaBH_4_ (0.8 equiv)	THF	−70	4	60%:12%:28%	67
4^b^	NaBH_4_ (0.8 equiv)	THF	25	2	67%:22%:11%	61
5^b^	NaBH_4_ (0.8 equiv)	THF	−30	2.5	70%:21%:2%	54
6	NaBH_3_CN (1 equiv)	THF	−20	2	ND^c^	
7	BH_3_ (1 equiv)	THF	−20	2	82%:14%:4%	71
8	BH_3_ (0.8 equiv)	THF	−20	2	81%:13%:6%	72
9	BH_3_ (0.6 equiv)	THF	−20	6	76%:14%:6%	69
10	BH_3_ (0.8 equiv)	THF	0	1	81%:17%:2%	66
11	BH_3_ (0.8 equiv)	THF	25	0.5	81%:15%:4%	69
12	BH_3_ (0.8 equiv)	MTBE	25	1	80%:11%:9%	76
13	BH_3_ (0.8 equiv)	2-MeTHF	25	1	80%:16%:4%	67
14	BH_3_ (0.8 equiv)	IPE^d^	25	1	85%:10%:4%	78
15	BH_3_ (0.8 equiv)	IPE^d^	0	1.5	87%:5%:1%	89
16	BH_3_ (0.8 equiv)	IPE^d^	−25	3	^e^	85

^a^Diastereoisomeric excess of **5** and **5’**; ^b^added AcOH (0.1 equiv) in the reaction; ^c^no products were detected; ^d^diisopropyl ether; ^e^**5**:**5’**:**4** 55%:4%:40%.

Following a procedure reported in the literature [14], the reduction of **4** was carried out with NaBH_4_ in THF at 25 °C for 1 h (Table 1, entry 1). However, the main product was proven to be the denaphthalenyloxy compound **5’’** by ^1^H NMR and MS while the desired sulfinamide **5** was obtained only in a yield of 28%. The amount of denaphthalenyloxy was greatly reduced when the reaction temperature was decreased to −30 °C (Table 1, entry 2), but no significant improvement was achieved by further decreasing the temperature (Table 1, entry 3). It was assumed that after reduction, the basicity resulting from NaBH_4_ might lead to the denaphthalenyloxylation. Therefore, AcOH was used as an additive in the reaction. The results showed that the denaphthalenyloxylation was almost negligible, but the diastereoselectivity was not good enough (Table 1, entry 5). Although mild reductant, NaBH_3_CN, was then applied in the reaction, no reaction took place (Table 1, entry 6). When BH_3_ was used to react with **4** at −20 °C for 2 h, the result was promising in terms of both yield and de (Table 1, entry 7). The data of entries 7–11 indicated that 0.8 equiv of the reductant BH_3_ was sufficient and the optimized temperature was 0–25 °C when the reaction was carried out in THF. When other solvents were tested (Table 1, entries 12–14), it was found that diisopropyl ether gave the best result. Finally, the reaction was performed with 0.8 equiv BH_3_ in isopropyl ether at 0 °C for 1.5 h (Table 1, entry 15) and the de of the crude product was 89%. Compound **5** was isolated in pure form from the crude reaction mixture by recrystallization from 10% ethyl acetate/*n*-heptane in 79.2% yield with 99.0% de.

Then purified **5** was hydrolyzed in methanol with HCl/EtOH solution at room temperature and dissociated with NaHCO_3_ to give the primary amine **6** in 90.0% yield. The reductive amination of **6** under Eschweiler–Clarke conditions furnished (*S*)-dapoxetine **7** with excellent enantiopurity (99.3% ee) in 74.7% yield. After salt formation and recrystallization, the target compound **1** was obtained. The optical rotation value of compound **1** was consistent with that previously reported [15], which confirmed that the *S*-enantiomer of dapoxetine hydrochloride was synthesized successfully by using this route.

## Conclusion

In summary, a novel and stereoselective synthesis of dapoxetine hydrochloride starting from commercially available 3-(naphthalen-1-yloxy)-1-phenylpropan-1-one in five linear steps (33.5% overall yield) via introduction of the chiral auxiliary (*S*)-*tert*-butanesulfinamide was developed. This method was easy to perform and both the purity and yield of the product were excellent.

## Experimental

All solvents and reagents were of reagent grade and used without further purification. ^1^H and ^13^C NMR spectra were recorded using a Bruker 400 MHz spectrometer with TMS as an internal standard. HPLC analyses were recorded with on a Dionex Ultimate 3000 chromatograph and chiral HPLC analyses were recorded with an Agilent 1100 Series spectrometer.

**Preparation of (*S*)-2-methyl-*N*-(3-(naphthalen-1-yloxy)-1-phenylpropylidene)propane-2-sulfinamide (4):** To a solution of **3** (30 g, 0.11 mol) and (S)-*tert*-butanesulfinamide (14.7 g, 0.12 mol) in THF (300 mL), Ti(OEt)_4_ (61.8 g, 0.22 mol) was added under N_2_ atmosphere and the mixture was refluxed at 65 °C for about 8 h. Upon completion (as determined by TLC), the reaction mixture was first cooled to rt and then quenched with ethyl acetate (300 mL) and brine (300 mL), then stirred for 1 h, filtered, and the filtrate was washed with brine and dried over Na_2_SO_4_ and concentrated. The residue was purified via flash chromatography with petrol ether/ethyl acetate (20:1) to give **4** as a pale yellow solid. Yield 32.3 g (78.3%); mp 49–51 °C; 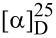 −10 (*c* 0.8, CDCl_3_); ^1^H NMR (400 MHz, CDCl_3_/TMS) δ 8.10 (d, *J* = 8 Hz, 1H), 7.79 (d, *J* = 8 Hz, 1H), 7.51–7.28 (m, 8H), 6.81 (d, *J* = 7.6 Hz, 1H), 4.62–4.49 (m, 2H), 4.02–3.79 (m, 2H), 1.39 (s, 9H); ^13^C NMR (100 MHz, CDCl_3_/TMS) δ 197.7, 154.4, 137.0, 134.6, 133.3, 128.7, 128.2, 127.4, 126.3, 125.8, 125.7, 125.1, 122.0, 120.5, 105.0, 77.3, 77.0, 76.6, 63.9, 38.2; HRMS (ES^+^) *m*/*z*: [M + Na]^+^ calcd for C_23_H_25_NO_2_NaS, 402.1504; found, 402.1493.

**Preparation of (*S*)-2-methyl-*N-*((*S*)-3-(naphthalen-1-yloxy)-1-phenylpropyl)propane-2-sulfinamide (5):** To a suspension of **4** (20 g**,** 53 mmol) in diisopropyl ether (300 mL), BH_3_/THF (10 mL, 42.2 mmol) was added dropwise at −5 to 0 °C. After this addition, the reaction mixture was stirred for 1.5 h. The color of the reaction changed from yellow to white and TLC showed the complete consumption of **4**. Then, ethyl acetate (200 mL) and water (100 mL) were added and the mixture was stirred for 5 min and then separated. The organic phase was washed with brine, dried over Na_2_SO_4_, and filtered and concentrated to obtain the crude product. The crude product was crystallized from an *n*-heptane/ethyl acetate mixture (9:1) to get pure **5** as an off-white solid. Yield 15.9 g (79.2%); mp 60–61 °C; 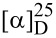 65.8 (*c* 1, CDCl_3_); de 99.0%; ^1^H NMR (400 MHz, CDCl_3_/TMS) δ 8.20 (d, *J* = 8.4 Hz, 1H), 7.79 (d, *J* = 8.4 Hz, 1H), 7.51–7.28 (m, 8H), 6.67 (d, *J* = 7.6 Hz, 1H), 4.80 (m, 1H), 4.15 (m, 1H), 4.02 (m, 1H), 3.58 (d, NH), 2.66 (m, 1H), 2.35 (m, 1H), 1.22 (s, 9H); ^13^C NMR (100 MHz, CDCl_3_/TMS) δ 154.5, 141.9, 134.6, 128.9, 128.1, 127.4, 127.2, 126.4, 125.7, 125.3, 122.0, 120.4, 104.6, 94.5, 77.3, 77.2, 77.0, 76.7, 64.7, 56.8, 55.9, 36.5, 22.6; HRMS (ES^+^) *m*/*z*: [M + H]^+^ calcd for C_23_H_28_NO_2_S, 382.1841; found, 382.1842.

**Preparation of (*S*)-3-(naphthalen-1-yloxy)-1-phenylpropan-1-amine (6):** To a solution of **5** (12 g, 31.5 mmol) dissolved in methanol (60 mL), 28% HCl/EtOH (9 mL, 63 mmol) was added at 10–20 °C. The mixture was stirred for 1 h at room temperature. Then, the mixture was concentrated and the obtained crude residue was resuspended with MTBE (70 mL) to give pure hydrochloride **6.** The solid was suspended in DCM (50 mL), and saturated aqueous NaHCO_3_ solution (15 mL) was added and stirred until the mixture was no longer turbid. The organic phase was washed with brine, dried over Na_2_SO_4_, and filtered and concentrated to give **6** as a pale yellow oil. Yield 7.4 g (90.0%); 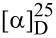 66.1 (*c* 0.3, CDCl_3_); ^1^H NMR (400 MHz, CDCl_3_/TMS) δ 8.26 (d, *J* = 8.4 Hz, 1H), 7.79 (d, *J* = 8.4 Hz, 1H), 7.51–7.28 (m, 8H), 6.75 (d, 1H), 4.35 (m, 1H), 4.23 (m, 1H), 4.11 (m, 1H), 2.34 (m, 2H); ^13^C NMR (100 MHz, CDCl_3_/TMS) δ 154.6, 145.0, 134.6, 128.7, 127.4, 127.4, 126.4, 126.3, 125.8, 125.1, 122.0, 120.3, 104.8, 77.3, 77.0, 76.7, 65.5, 53.7, 38.5; MS (ES^+^) *m*/*z*: 278 [M + H]^+^.

**Preparation of dapoxetine ((*S*)-*N,N*-dimethyl-3-(naphthalen-1-yloxy)-1-phenylpropan-1-amine, 7):** To a 50 mL round-bottomed flask, **6** (6 g, 21.6 mmol), 98% HCOOH (3.9 mL, 54.1 mmol) and an aqueous solution of 30% formaldehyde (9.7 mL, 108 mmol) were added at room temperature. The reaction mixture was heated to 85 °C for 8 h and quenched with saturated aqueous NaHCO_3_ solution (pH ≈8). The aqueous layer was extracted with EtOAc (20 mL, twice). The organic phase was washed with water, brine, dried over Na_2_SO_4_ and concentrated. The residue was purified by flash chromatography to afford dapoxetine as a colorless oil. Yield 4.95 g (74.7%); chiral purity (HPLC): 99.63%; ^1^H NMR (400 MHz, CDCl_3_/TMS) δ 8.26 (d, *J* = 9.2 Hz, 1H), 7.79 (d, *J* = 9.2 Hz, 1H), 7.47 (m, 2H), 7.35–7.28 (m, 7H), 6.67 (d, *J* = 8 Hz, 1H), 4.13–4.08 (m, 1H), 3.98–3.94 (m, 1H), 3.64–3.60 (m, 1H), 2.67–2.62 (m, 1H), 2.33–2.31 (m, 1H), 2.29 (s, 6H); ^13^C NMR (100 MHz, CDCl_3_/TMS) δ 154.7, 134.5, 128.6, 128.2, 127.4, 126.3, 125.8, 125.0, 122.0, 120.1, 104.7, 77.3, 77.0, 76.6, 67.8, 65.7, 42.7, 33.0; MS (ES^+^) *m*/*z*: 306 [M + H]^+^.

**Preparation of dapoxetine hydrochloride (1):** To a solution of **7** (3 g, 9.8 mmol) dissolved in diisopropyl ether (30 mL), 28% HCl/EtOH (1.3 mL, 1.2 equiv) was added dropwise at room temperature. A white solid was precipitated and filtered to obtain the crude **1** (2.8 g). The solid was recrystallized from isopropyl alcohol/*n*-hexane (9 mL:8 mL) to give the product. Yield 2.7 g (80.4%); mp 178–180 °C; 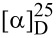 126.4 (*c* 1, methanol) [lit. [15] mp 180–184 °C; 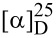 131.7 (*c* 1, methanol)]; chiral purity (HPLC): >99% ee; ^1^H NMR (400 MHz, DMSO-*d*_6_/TMS) δ 11.21 (brs, 1H, HCl), 8.06–6.73 (m, 12H), 4.71 (m, 1H), 4.11 (m, 1H), 3.75 (m, 1H), 2.95 (m, 1H), 2.92 (s, 3H), 2.73 (m, 1H), 2.58 (s, 3H); ^13^C NMR (100 MHz, DMSO-*d*_6_/TMS) δ 153.7, 134.0, 132.5, 129.9, 129.7, 129.0, 127.4, 126.5, 126.1, 125.2, 124.9, 121.8, 120.2, 105.1, 67.3, 64.6, 41.4, 40.3, 40.1, 39.9, 39.7, 39.5, 39.3, 39.0, 29.6; MS (ES^+^) *m*/*z*: 306 [M + H]^+^.

## Supporting Information

File 1 ^1^H NMR, ^13^C NMR and ESIMS spectra of compounds **1**, **4**, **5**, **5”**, **6** and **7** and chiral HPLC diagrams of **1** and **7**.
