# Supplementary material for: A novel and practical asymmetric synthesis of dapoxetine hydrochloride
Source: Beilstein J Org Chem. 2015 Dec 17;11:2641–5. doi: 10.3762/bjoc.11.283 (PMC4685759; doi:10.3762/bjoc.11.283)
Supplement: File 1 — 1H NMR, 13C NMR and ESIMS spectra of compounds 1, 4, 5, 5”, 6 and 7 and chiral HPLC diagrams of 1 and 7. [file Beilstein_J_Org_Chem-11-2641-s001.pdf]

Supporting Information

for

A novel and practical asymmetric synthesis of dapoxetine hydrochloride

Yijun Zhu[1,2], Zhenren Liu[1], Hongyan Li[1], Deyong Ye[2,*] and Weicheng Zhou[1,*]

Address: [1]State Key Lab of New Drug & Pharmaceutical Process, Shanghai Key Lab of Anti-Infectives, State Institute of Pharmaceutical Industry, No.285, Gebaini Rd, Shanghai 201203, China and [2]School of Pharmacy, Fudan University, No.826, Zhangheng Rd., Shanghai 201203, China

Email: Deyong Ye - dyye@shmu.edu.cn;

Weicheng Zhou - zhouweicheng58@163.com

* Corresponding author

^{1}H NMR, ^{13}C NMR and ESIMS spectra of compounds 1, 4, 5, 5”, 6 and 7 and chiral HPLC diagrams of 1 and 7

Table of contents

^{1}H NMR and ^{13}C NMR spectra of compound **4** S3

HRMS of **4** and ^{1}H NMR spectrum of **5** S4

^{13}C NMR and HRMS spectra of **5** S5

^{1}H NMR and ESIMS spectra of compound **5”** S6

^{1}H NMR and ^{13}C NMR spectra of compound **6** S7

ESIMS of **6** and ^{1}H NMR spectrum of **7** S8

^{13}C NMR and ESIMS spectra of **7** S9

^{1}H NMR and ^{13}C NMR spectra of compound **1** S10

ESI-MS spectra of compound **1** S11

Chiral HPLC of **7** S12

Chiral HPLC of **1** S13

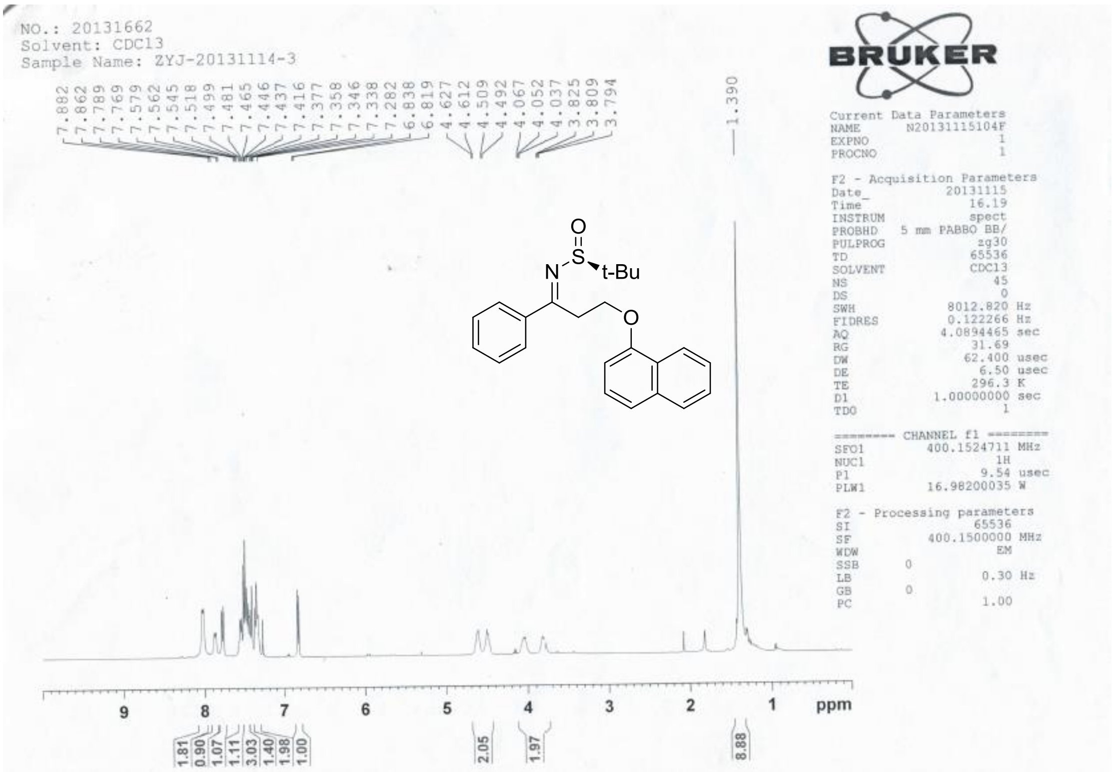

^{1}H NMR spectrum of compound **4**

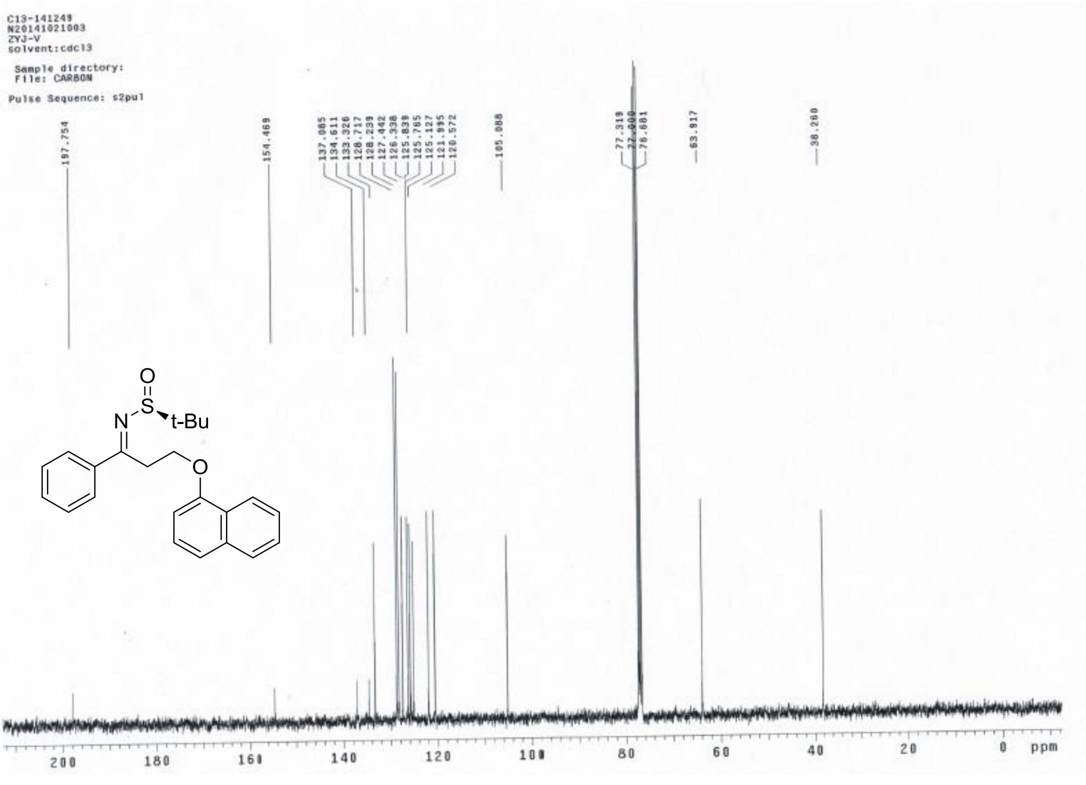

^{13}C NMR spectrum of compound **4**

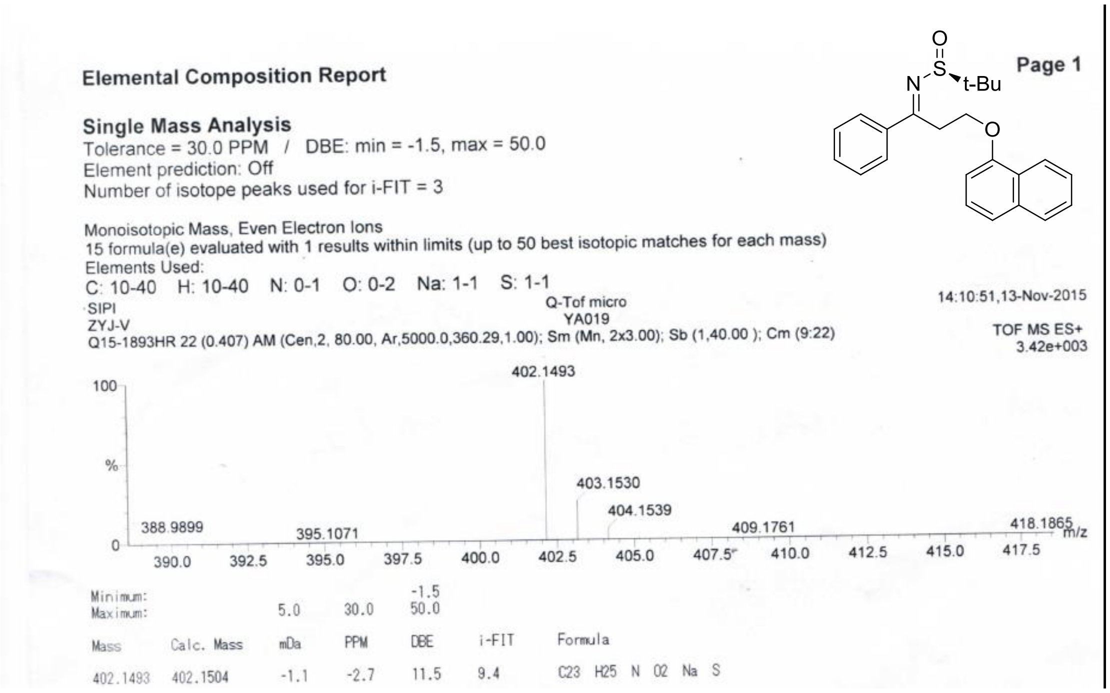

HRMS spectrum of compound **4**

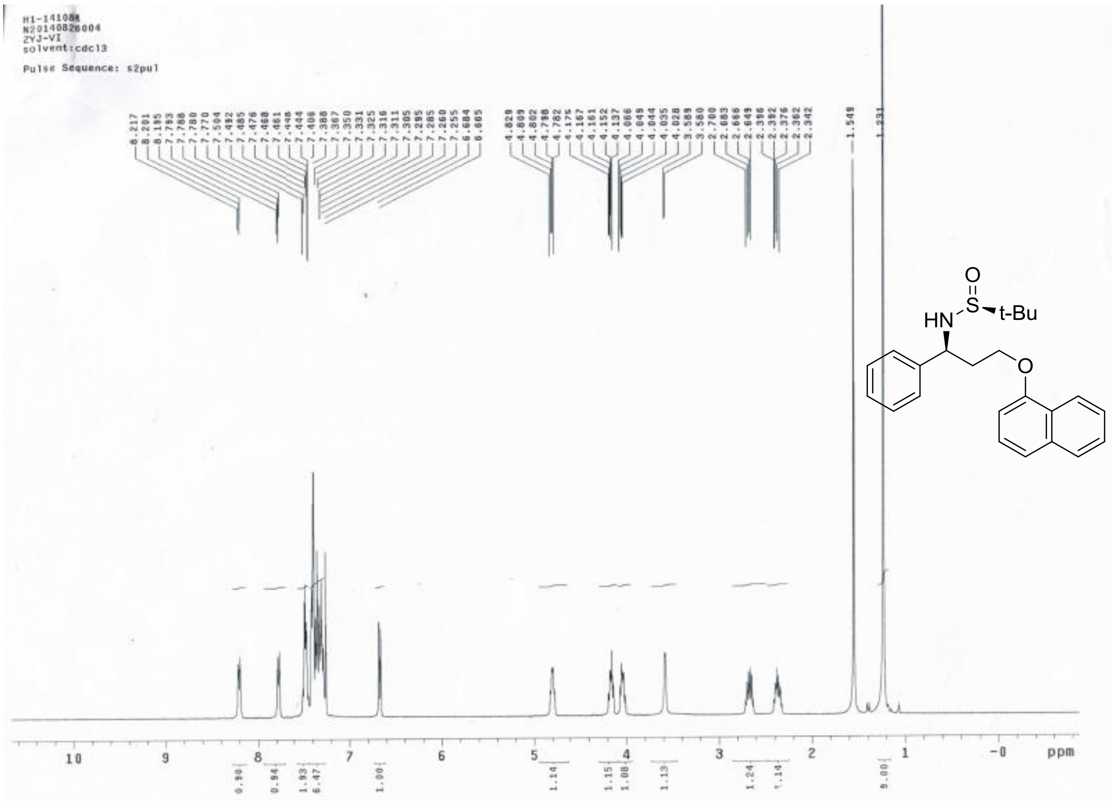

^{1}H NMR spectrum of compound **5**

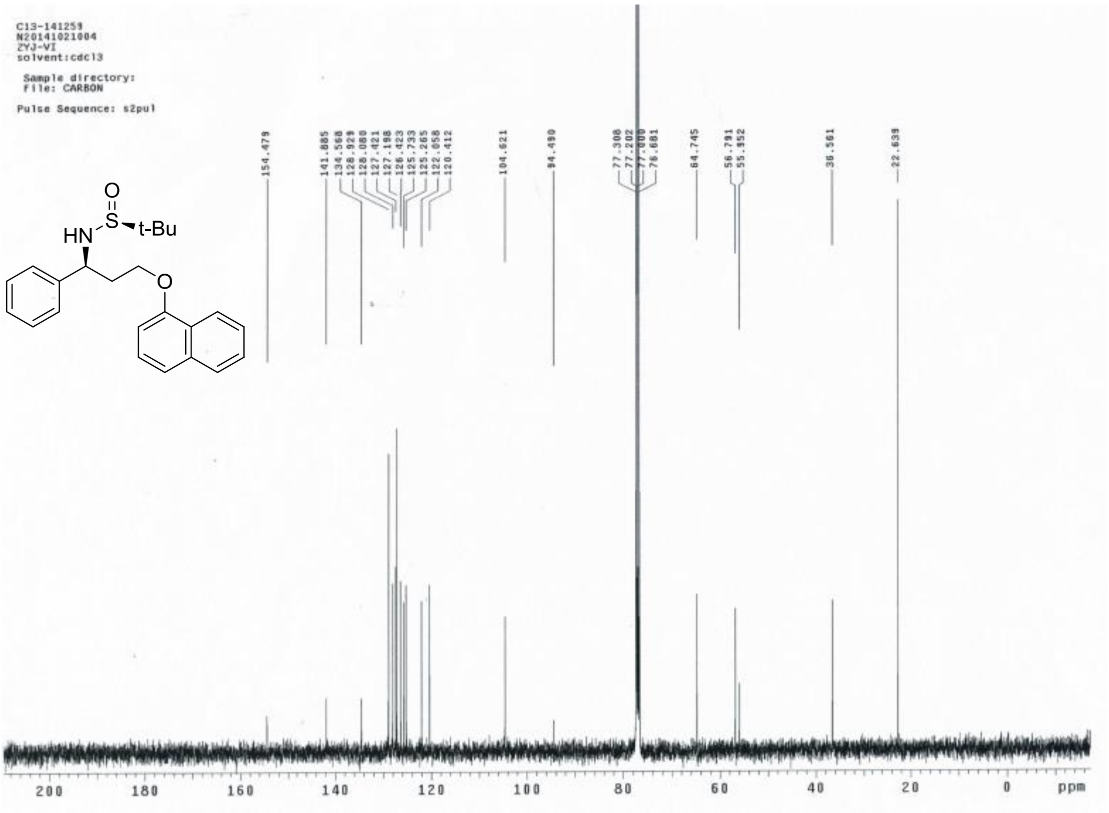

^{13}C NMR spectrum of compound **5**

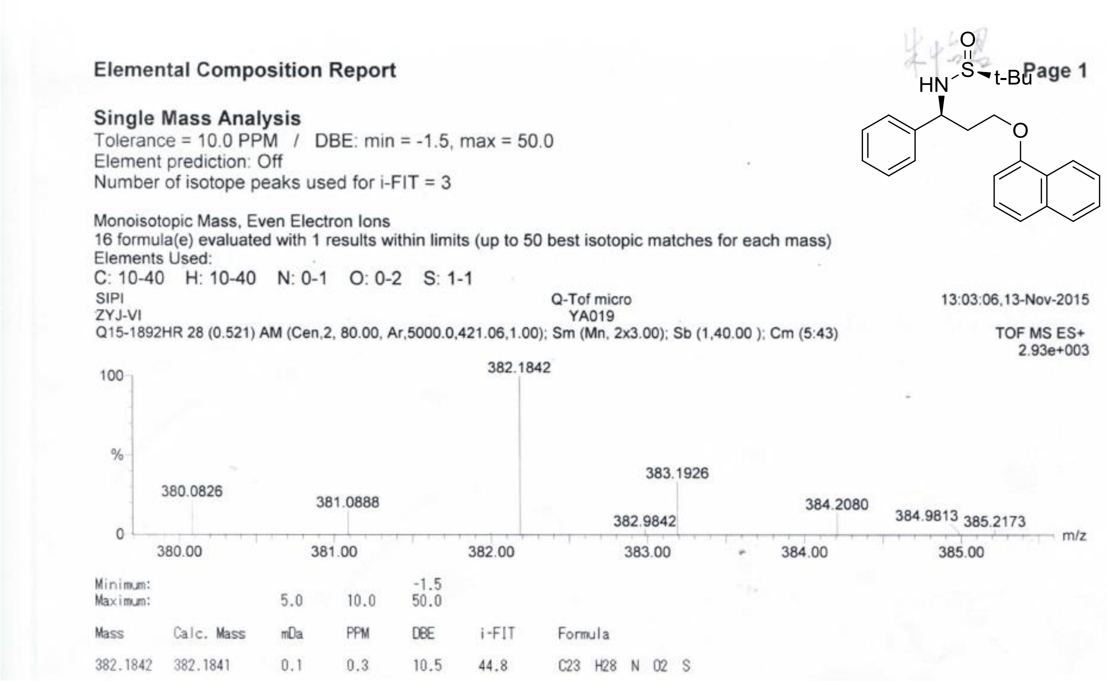

HRMS spectrum of compound **5**

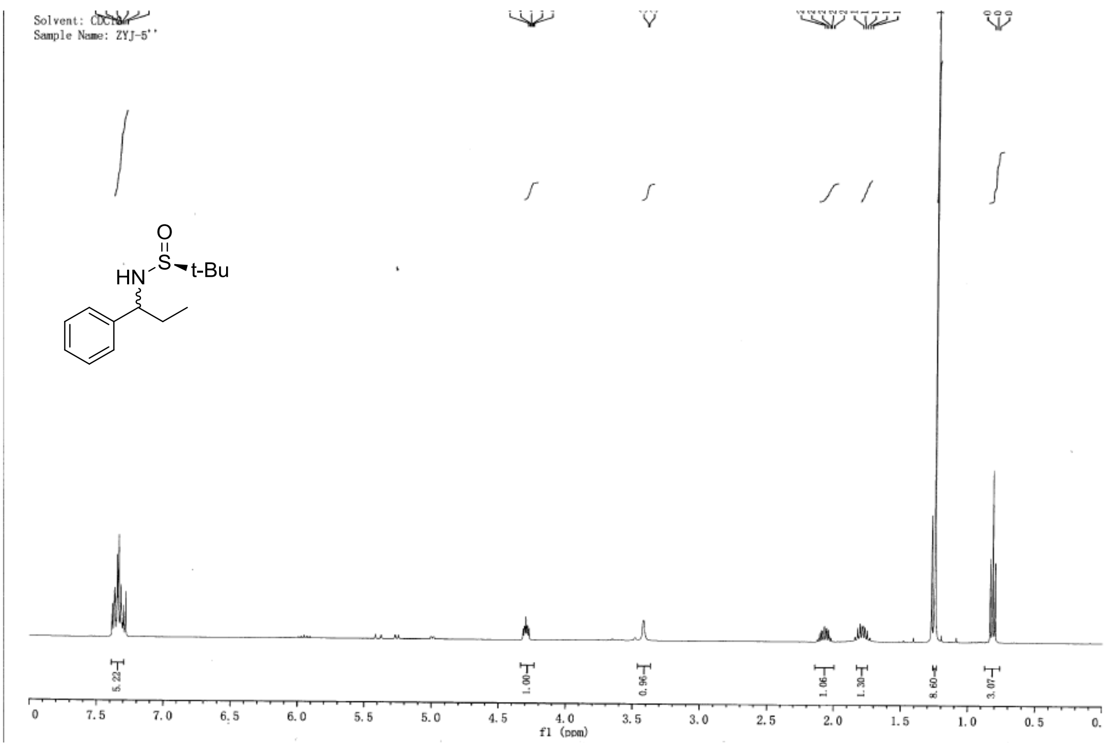

^{1}H NMR spectrum of compound **5”**

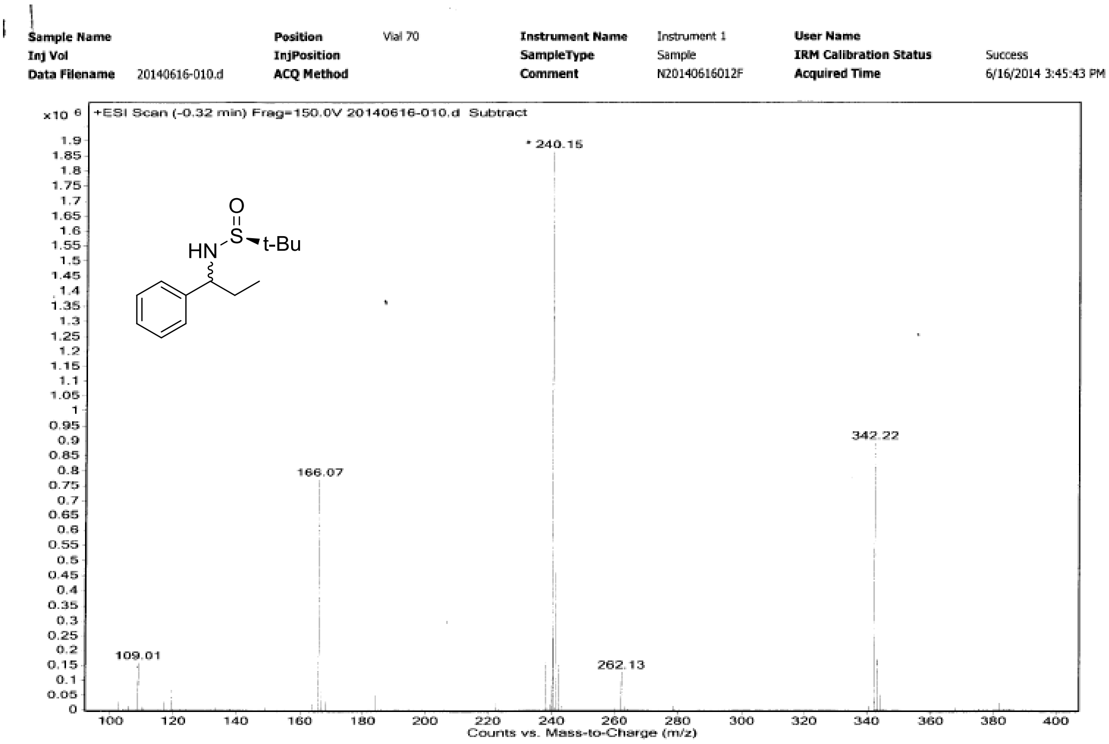

ESIMS spectrum of compound **5”**

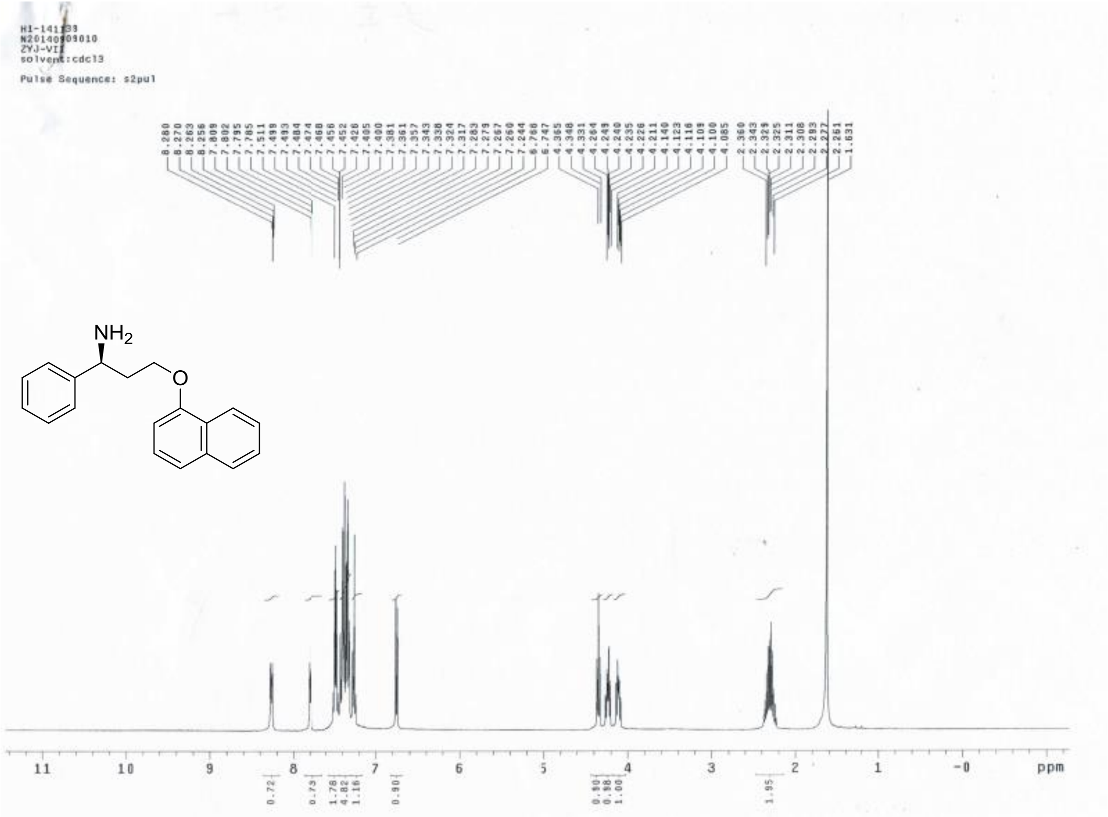

^{1}H NMR spectrum of compound **6**

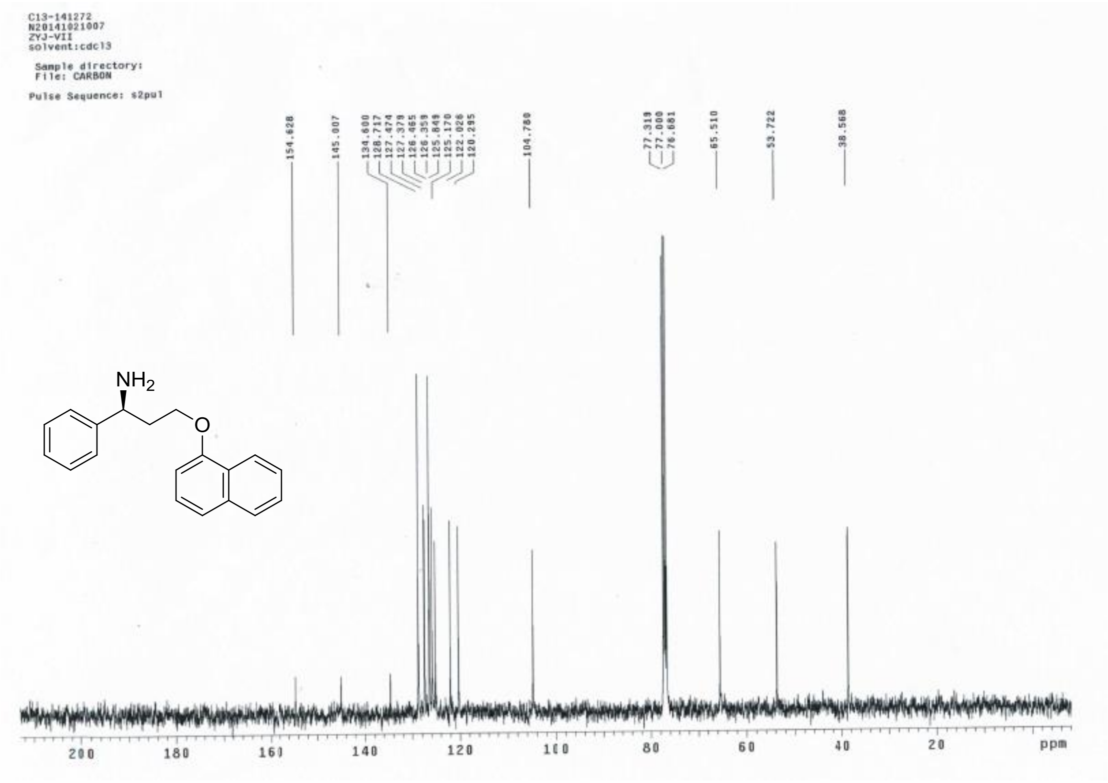

^{13}C NMR spectrum of compound **6**

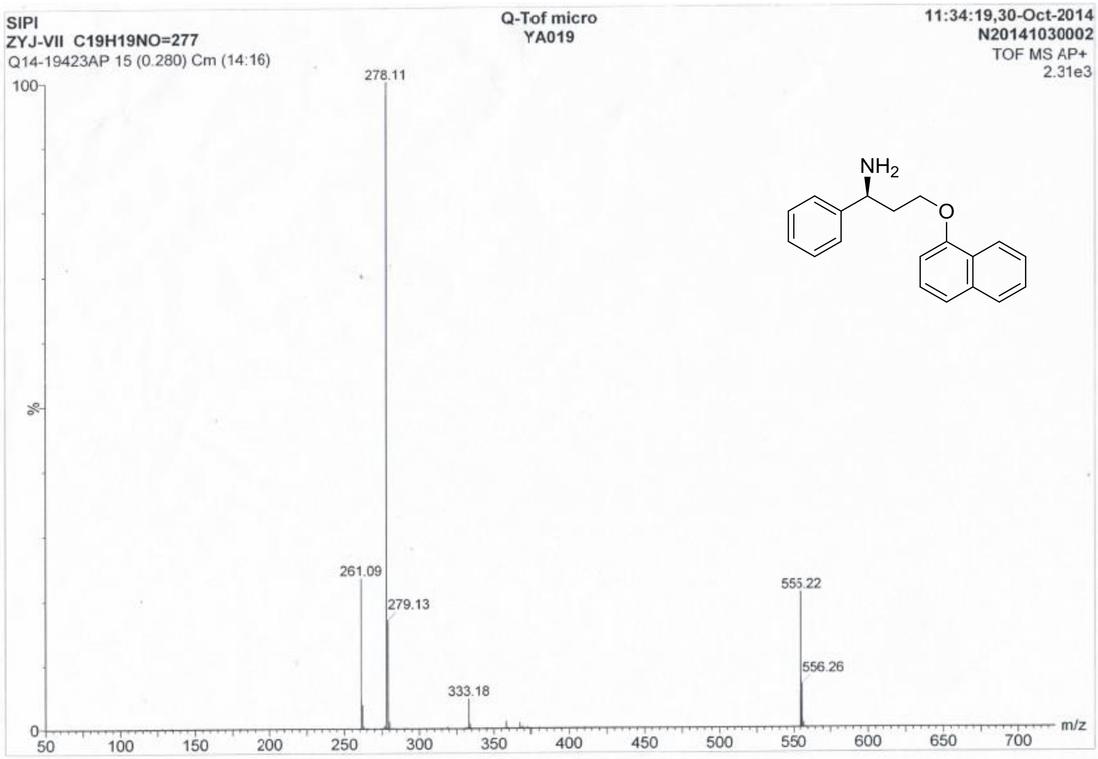

ESIMS spectrum of compound **6**

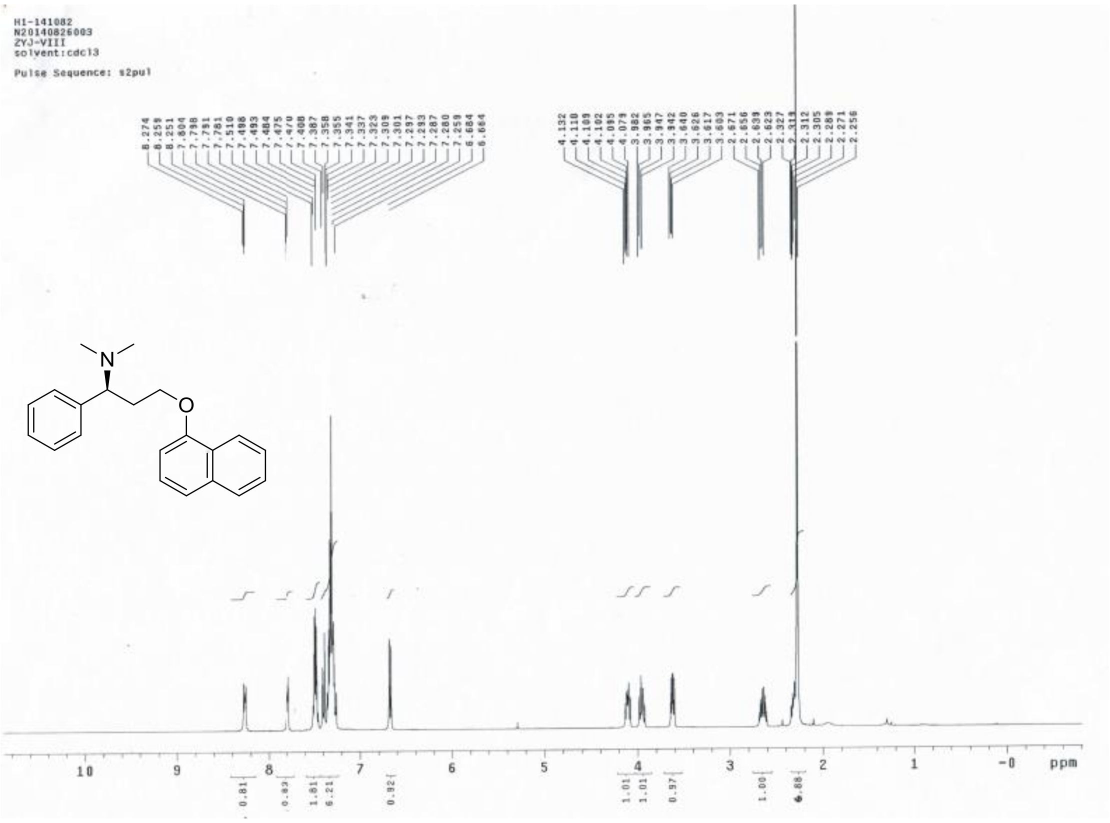

^{1}H NMR spectrum of compound **7**

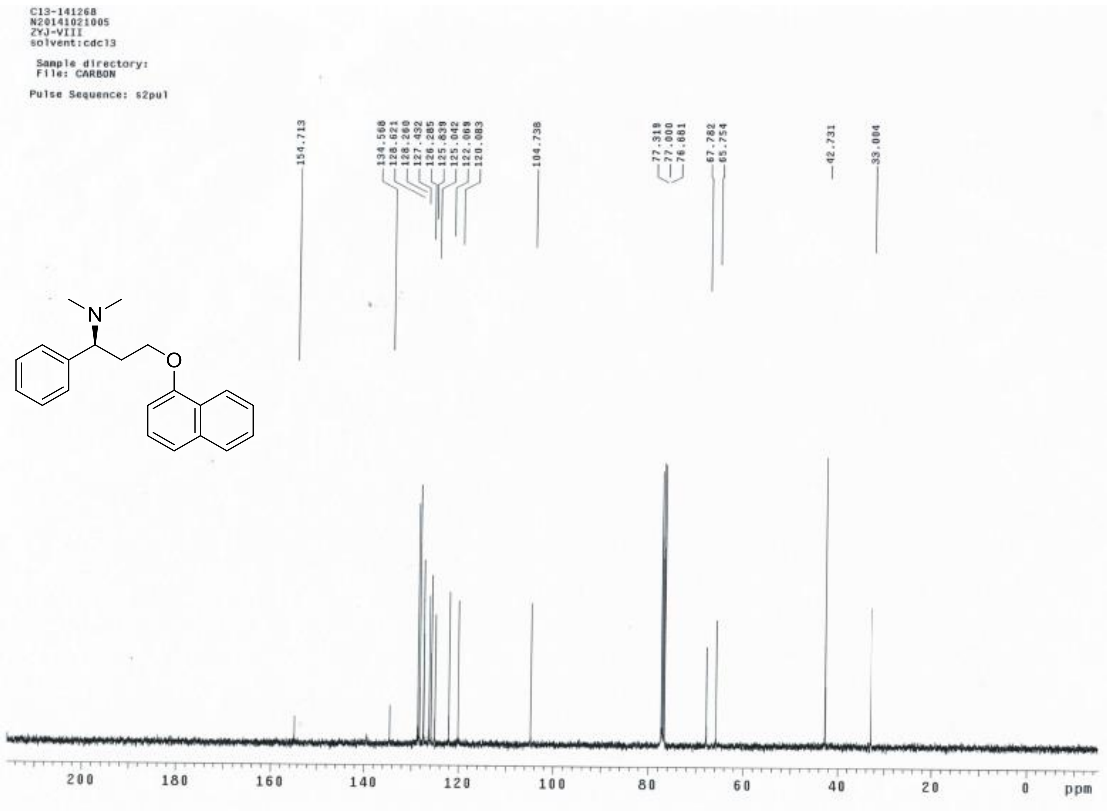

^{13}C NMR spectrum of compound 7

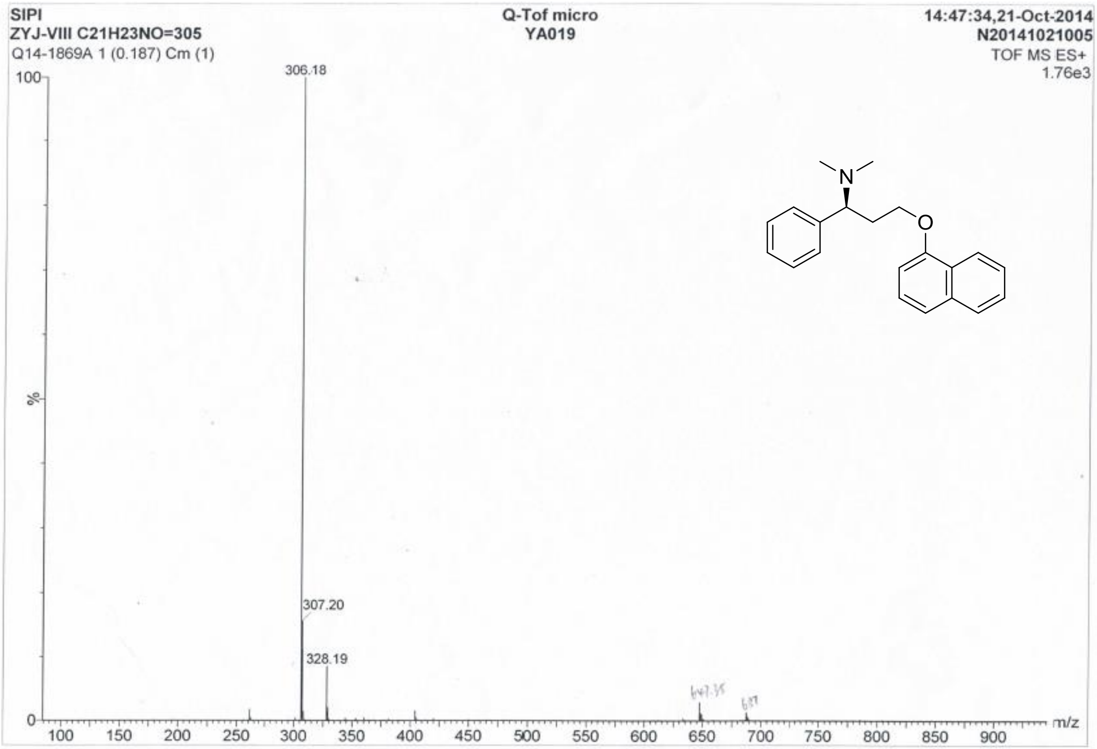

ESIMS spectrum of compound 7

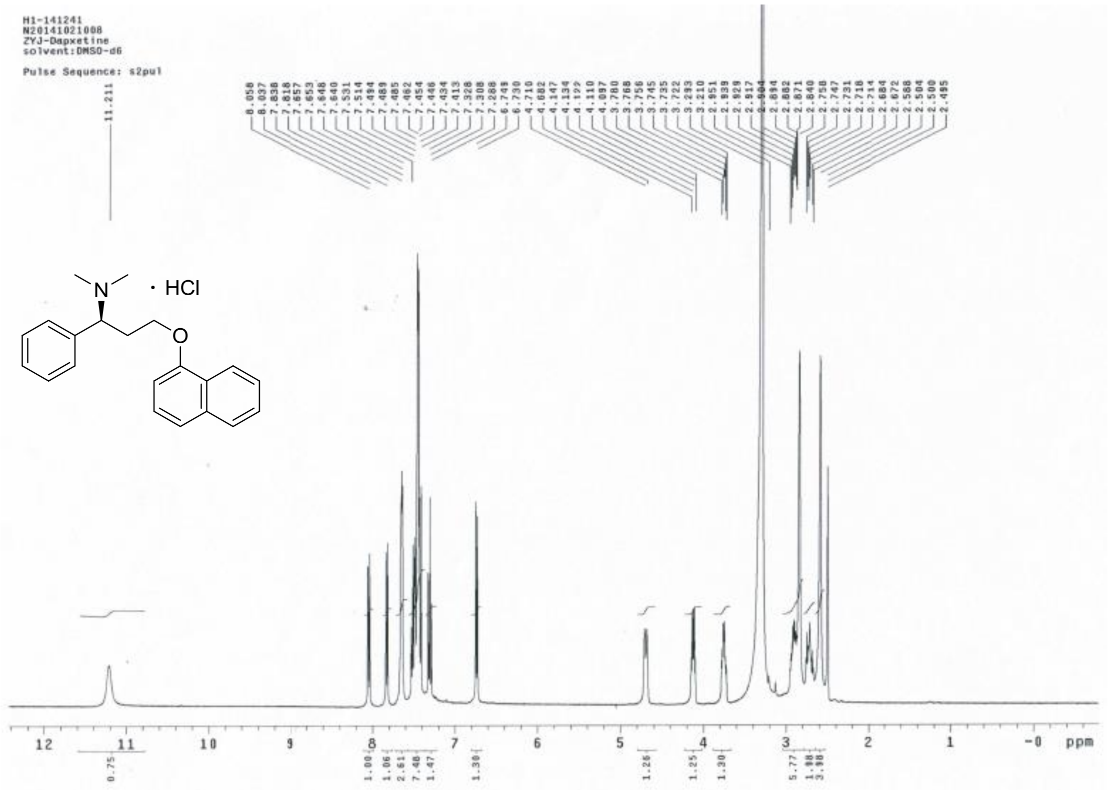

^{1}H NMR spectrum of compound **1**

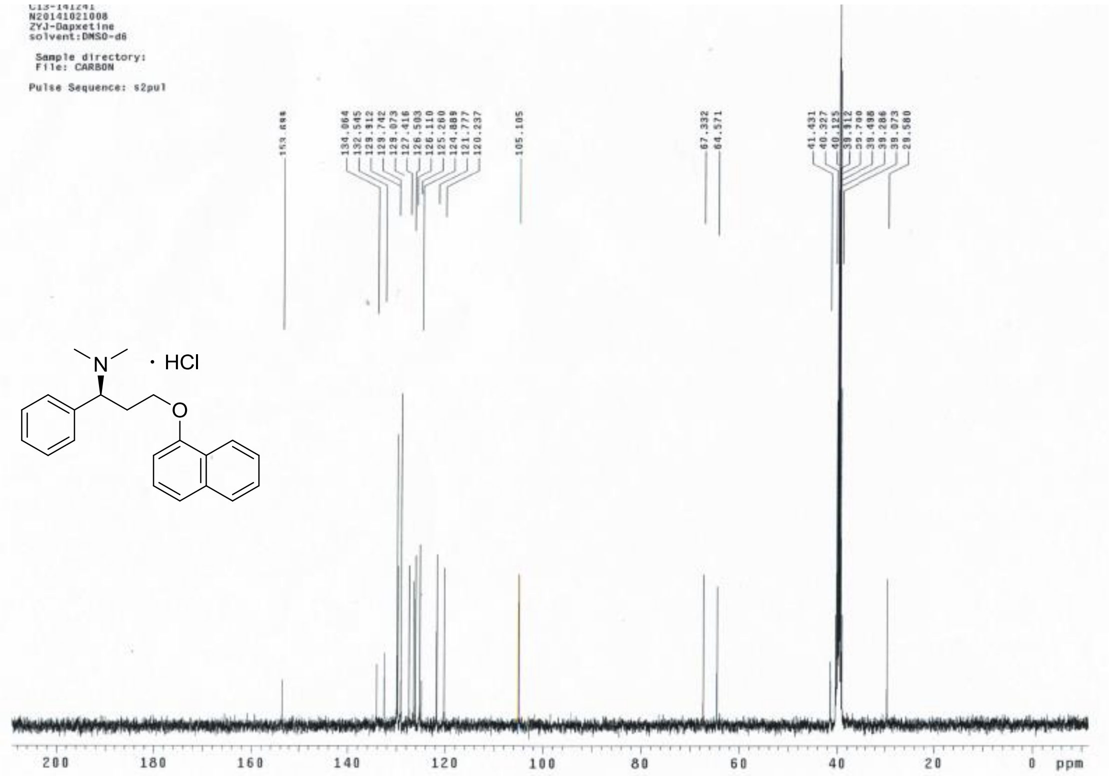

^{13}C NMR spectrum of compound **1**

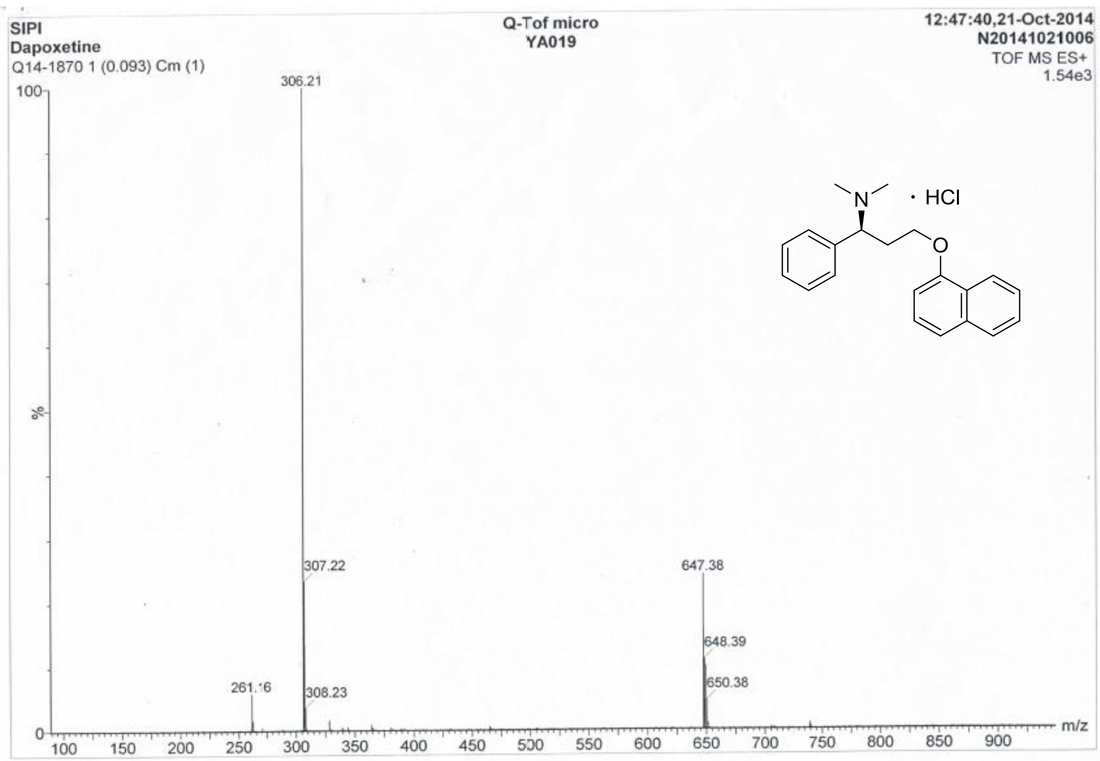

ESIMS spectrum of compound **1**

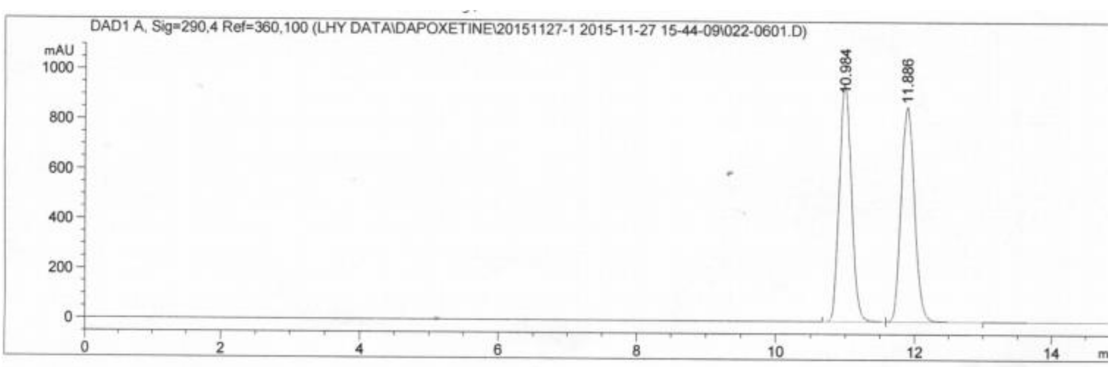

Area Percent Report

Sorted By : Signal
Multiplier : 1.0000
Dilution : 1.0000
Use Multiplier & Dilution Factor with ISTDs

Signal 1: DAD1 A, Sig=290,4 Ref=360,100

Peak #	RetTime [min]	Type	Width [min]	Area [mAU*s]	Height [mAU]	Area %
1	10.984	BB	0.1886	1.14817e4	952.94586	49.9049
2	11.886	BB	0.2081	1.15254e4	861.45459	50.0951

Chiral HPLC of **Racemic-7**

(modified after loading)

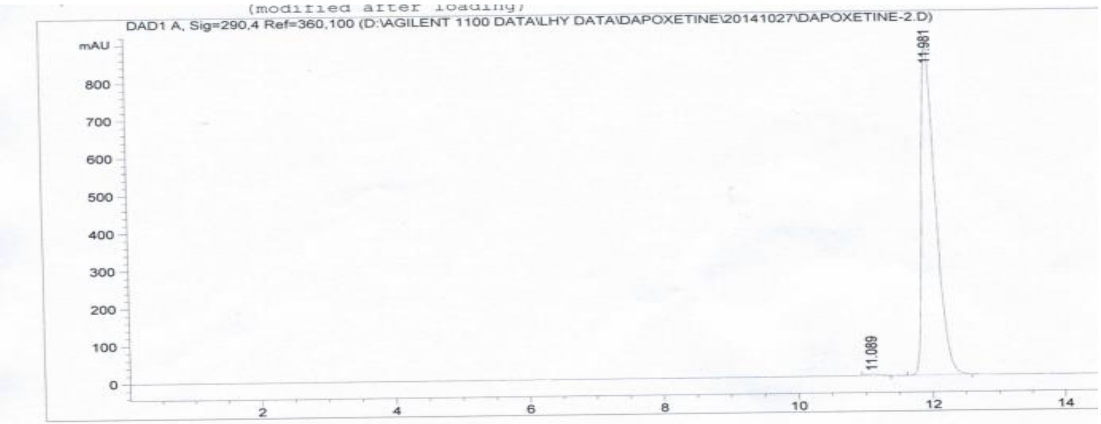

Area Percent Report

Sorted By : Signal
Multiplier : 1.0000
Dilution : 1.0000
Use Multiplier & Dilution Factor with ISTDs

Signal 1: DAD1 A, Sig=290,4 Ref=360,100

Peak #	RetTime [min]	Type	Width [min]	Area [mAU*s]	Height [mAU]	Area %
1	11.089	BB	0.1448	47.24658	4.21311	0.3667
2	11.981	BB	0.2249	1.28360e4	876.36298	99.6333

Chiral HPLC of **7**

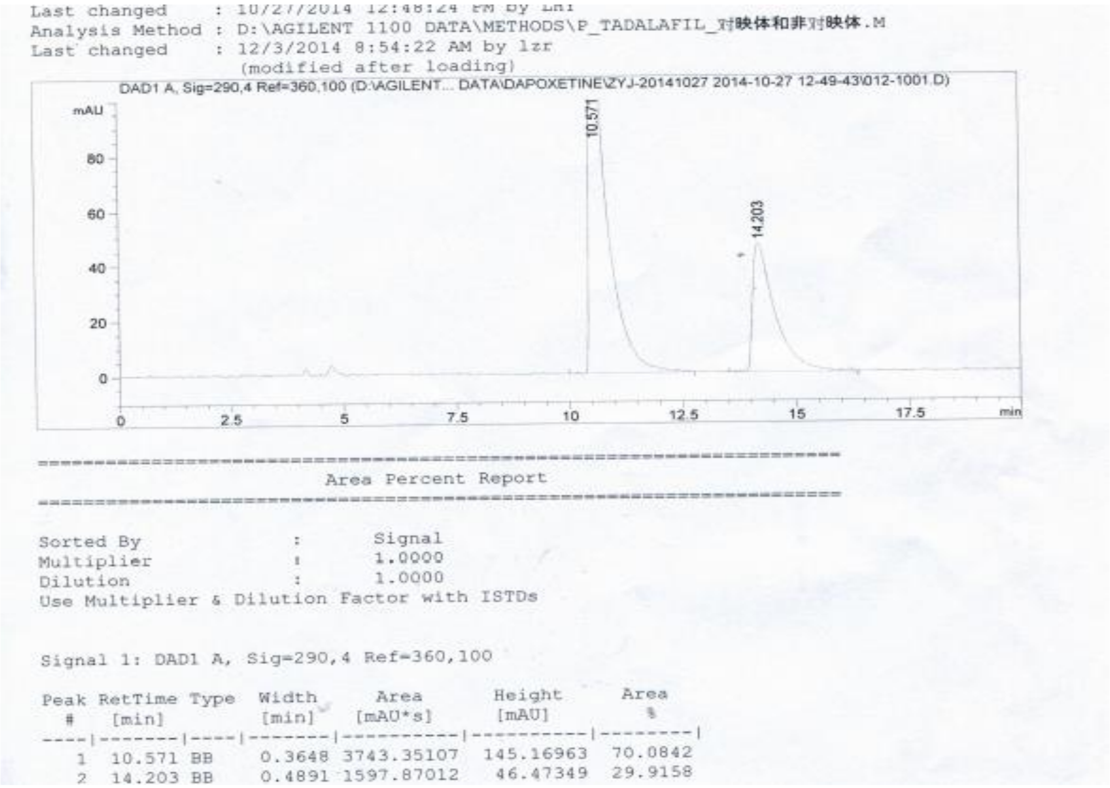

Last changed : 10/27/2014 12:48:24 PM by LHY
Analysis Method : D:\AGILENT 1100 DATA\METHODS\P_TADALAFIL_对映体和非对映体.M
Last changed : 12/3/2014 8:54:22 AM by lzr
(modified after loading)

Area Percent Report

Sorted By : Signal
Multiplier : 1.0000
Dilution : 1.0000
Use Multiplier & Dilution Factor with ISTDs

Signal 1: DAD1 A, Sig=290,4 Ref=360,100

Peak #	RetTime [min]	Type	Width [min]	Area [mAU*s]	Height [mAU]	Area %
1	10.571	BB	0.3648	3743.35107	145.16963	70.0842
2	14.203	BB	0.4891	1597.87012	46.47349	29.9158

Mixed Sample of (R)-**1** and (S)-**1**

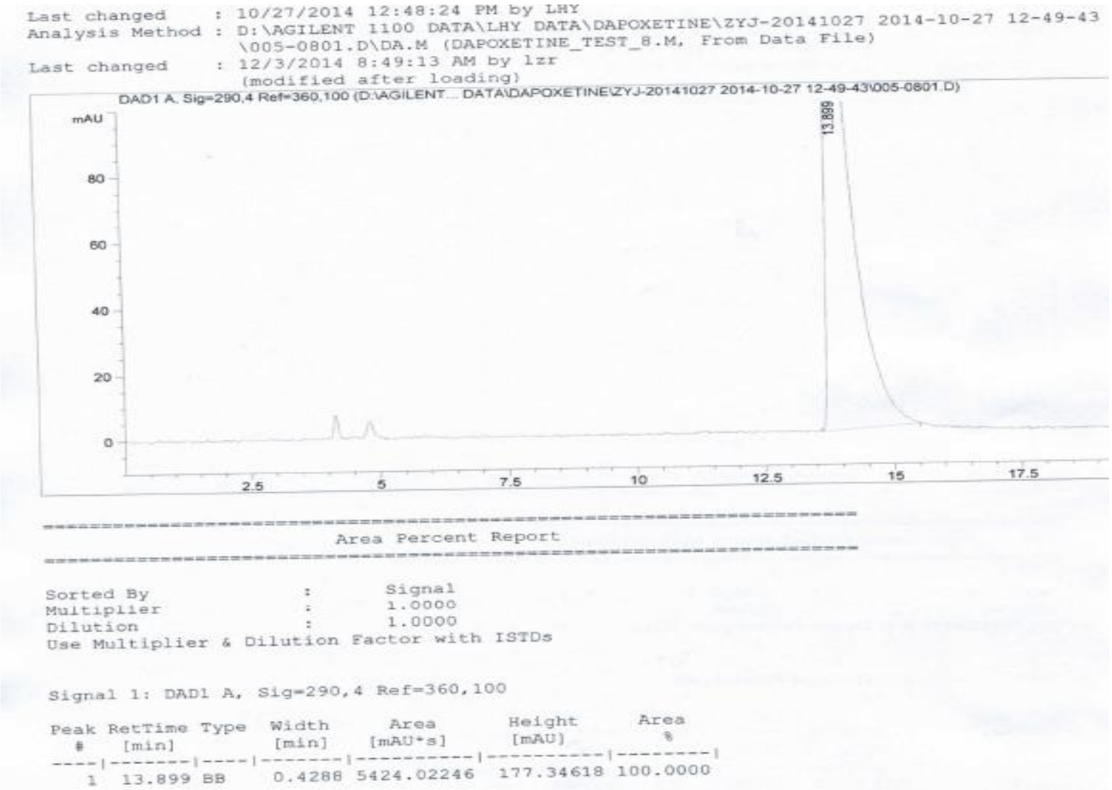

Last changed : 10/27/2014 12:48:24 PM by LHY
Analysis Method : D:\AGILENT 1100 DATA\LHY DATA\DAPOXETINE\ZYJ-20141027 2014-10-27 12-49-43 \005-0801.D\DA.M (DAPOXETINE_TEST_8.M, From Data File)
Last changed : 12/3/2014 8:49:13 AM by lzr
(modified after loading)

Area Percent Report

Sorted By : Signal
Multiplier : 1.0000
Dilution : 1.0000
Use Multiplier & Dilution Factor with ISTDs

Signal 1: DAD1 A, Sig=290,4 Ref=360,100

Peak #	RetTime [min]	Type	Width [min]	Area [mAU*s]	Height [mAU]	Area %
1	13.899	BB	0.4288	5424.02246	177.34618	100.0000

Chiral HPLC of **1**
